# Personal resources providing stress resistance of hospice medical workers

**DOI:** 10.1192/j.eurpsy.2021.1980

**Published:** 2021-08-13

**Authors:** M. Abdullaeva

**Affiliations:** Psychology, Lomonosov Moscow State University, Moscow, Russian Federation

**Keywords:** hospice medical workers, personal resources, stress resistance, professional burnout

## Abstract

**Introduction:**

The functional approach to the study of stress resistance allows us to distinguish two blocks of resources – the “active” one, which includes the analysis of the content, conditions, subject and means of labor, and the “personal” one, which considers values, motivation and the expressiveness of personal qualities that contribute to the stress resistance (Granek, Buchman, 2020; Cross, 2019; Powell et al., 2020; Hernández-Marrero, Fradique, 2019).

**Objectives:**

The objective of our work was to study the relationship between the personal and motivational characteristics of hospice employees with the different symptoms of professional burnout as an indicator of a reduced stress resistance.

**Methods:**

62 hospice medical employees with an average work experience of 4,5 years took part in the survey. They were asked to fill out questionnaires to diagnose the burnout symptoms, a motivational personality profile and to assess themselves by the personal semantic differential.

**Results:**

By the means of the procedure for determining the extreme groups (M ± σ), two groups of respondents were identified, which are characterized by different degrees of burnout symptoms. The results of the comparative analysis showed that the less advantaged respondents from the burnout perspective are focused on the life support, comfort, social status, which indicates a certain rationality in the choice of this job.

**Conclusions:**

The portrait of a professionally successful hospice employee includes an orientation towards communication, social and creative activity, which is complemented with independence, confidence and decisiveness – the features that allow carrying out their work in stressful conditions and mainly in uncertain situations.

**Disclosure:**

No significant relationships.

